# Effectiveness of Physical Activity in the Management of Nonspecific Low Back Pain: A Systematic Review

**DOI:** 10.3390/medicina60122065

**Published:** 2024-12-16

**Authors:** Alexandra Alonso-Sal, José Luís Alonso-Perez, María Dolores Sosa-Reina, Juan Antonio García-Noblejas-Fernández, Viren Gul Balani-Balani, Giacomo Rossettini, Jorge Hugo Villafañe

**Affiliations:** 1 Department of Physiotherapy, Faculty of Sport Sciences, Universidad Europea de Madrid, 28670 Villaviciosa de Odón, Spain; joseluis.alonso@universidadeuropea.es (J.L.A.-P.); mariadolores.sosa@universidadeuropea.es (M.D.S.-R.); juanitogn2000@gmail.com (J.A.G.-N.-F.); vir.balanif@gmail.com (V.G.B.-B.); 2 Musculoskeletal Pain and Motor Control Research Group, Faculty of Sport Sciences, Universidad Europea de Madrid, 28670 Villaviciosa de Odón, Spain; 3 Department of Physiotherapy, Faculty of Health Sciences, Universidad Europea de Canarias, 38300 La Orotava, Spain; 4 Musculoskeletal Pain and Motor Control Research Group, Faculty of Health Sciences, Universidad Europea de Canarias, 38300 La Orotava, Spain; 5 One Life Center, Multidisciplinary Pain Treatment Center, 28925 Madrid, Spain

**Keywords:** nonspecific low back pain, exercise, biopsychosocial factors, physical health, inflammatory biomarkers

## Abstract

*Background and Objectives:* This systematic review evaluates the effectiveness of exercise interventions for managing nonspecific low back pain (NSLBP) and explores their impact on related biopsychosocial factors, physical health variables, and inflammatory biomarkers. *Materials and Methods*: A comprehensive search of five databases (PubMed, CINAHL, PEDro, SCOPUS, Cochrane Library) was conducted, covering studies from 2019 to 2024. Fifteen randomized controlled trials involving 1338 participants aged 18 to 65 years with NSLBP were included. Data extraction and quality assessment were performed independently by two reviewers using the PEDro scale, and risk of bias was evaluated using the Cochrane Risk-of-Bias tool (RoB 2.0). *Results*: Exercise significantly reduced pain intensity and improved biopsychosocial factors such as depression, disability, functionality, quality of life, and kinesiophobia. Additionally, it enhanced physical parameters like proprioception, muscle thickness, and physical performance. However, the review found insufficient evidence regarding the effects of exercise on inflammatory biomarkers in NSLBP patients. *Conclusions*: The findings suggest that physical exercise is an effective intervention for pain reduction and the improvement of overall health in NSLBP, though further research is needed to clarify its impact on inflammation.

## 1. Introduction

Low back pain (LBP) has been the leading cause of disability worldwide for over three decades, contributing to substantial personal suffering and socioeconomic burdens, with healthcare costs exceeding USD 100 billion annually in the United States alone [1,2]. LBP is a primary cause of activity limitation, work disability, and sick leave among adults, significantly impacting quality of life and productivity [3]. Clinically, LBP is defined as pain, tension, or muscle stiffness located below the costal margin and above the inferior gluteal folds, potentially with referred pain to the legs, and is typically associated with restricted and painful movement [4]. The condition affects up to 80% of individuals at least once during their lifetime, and its prevalence is increasing among children and adolescents, reaching approximately 39% among those aged 9 to 16 years [5]. Although 33% of LBP patients recover within three months, about 65% continue to experience symptoms after one year, progressing to chronic low back pain (CLBP) due to a complex interaction of nociceptive stimuli and other factors [6]. A significant proportion of LBP cases, over 85% in primary care settings, are classified as “nonspecific low back pain” (NSLBP), a term used when the pain cannot be attributed to a specific, identifiable pathology such as fractures, infections, or malignancies [7,8,9]. The global burden of LBP is expected to increase, with the population of adults aged 60 years or older with LBP projected to double from its 2015 size, reaching 2.1 billion by 2050 [6].

Several risk factors contribute to the onset and recurrence of NSLBP, including demographic factors (such as obesity and menopause in women), psychological factors (such as anxiety and depression), and clinical factors (such as concurrent pain in other body regions or disc space narrowing) [10]. Central sensitization, characterized by an enhanced response to nociceptive stimuli, is thought to play a key role in the development and persistence of NSLBP. This may be mediated by inflammatory biomarkers such as interleukin-1 beta (IL-1β), interleukin-6 (IL-6), and tumor necrosis factor-alpha (TNF-α), which have been associated with the severity and presence of NSLBP [11,12]. Additional inflammatory markers, including C-reactive protein (CRP), growth differentiation factor 15 (GDF-15), and transforming growth factor-beta (TGF-β), have also been linked to NSLBP [13]. Moreover, muscle deconditioning, particularly isometric and isokinetic weakness of the lumbar musculature, is commonly observed in individuals with NSLBP [14].

NSLBP can manifest as chronic pain, experienced continuously, or as recurrent pain, occurring in cycles. Acute low back pain (ALBP) is characterized by the onset of symptoms over months or years, often leading to biomechanical impairments such as altered gait patterns, reduced walking speed, and impaired coordination [5]. Conservative treatment, including physiotherapy, has been demonstrated to be as effective as surgical intervention for many patients with LBP [15].

Exercise therapy, in particular, is recommended as a first-line treatment for both acute and chronic NSLBP due to its cost-effectiveness and its ability to improve quality-adjusted life years [16]. Evidence from European guidelines and the National Institute for Health and Care Excellence (NICE) suggests that maintaining physical activity and engaging in regular exercise can reduce the risk of NSLBP and enhance recovery [17,18]. Exercise is known to strengthen the lumbar musculature and may reduce the presence of inflammatory biomarkers, thus providing a multifaceted benefit for patients with NSLBP [19].

Given the extensive evidence supporting the use of exercise in the management of NSLBP and its effects on pain-related variables, this systematic review aims to synthesize the latest research on the efficacy of physical activity in patients with NSLBP. Additionally, it seeks to explore the effects of physical exercise on inflammatory biomarkers, psychosocial factors, and physical conditioning variables in this population.

## 2. Materials and Methods

This study was conducted following a systematic protocol based on the standards of the 2020 PRISMA (Preferred Reporting Items for Systematic Reviews and Meta-Analyses) statement. This guide consists of 27 items grouped into 7 sections aimed at improving the documentation and transparency of studies related to healthcare interventions. The goal of using the PRISMA guide is to ensure that the review includes adequate and complete information clearly and transparently. The authors highlight the usefulness of the PRISMA guide in planning and writing reviews, facilitating the collection and presentation of all relevant information based on available scientific evidence.

### 2.1. Search Strategy

The systematic search for the included articles was carried out between January and March 2024 by two independent reviewers (J.G.N. and V.B.), who used the following databases: PubMed, CINAHL, PEDro, SCOPUS, and Cochrane Library. The search considered the following free terms in English: “nonspecific low back pain”, “senior fitness test”, “IPAQ-SF”, “International Physical Activity Questionnaire Short Form”, “abdominal muscles”, “Core”, “body mass index”, “inflammatory biomarkers”, “immune inflammatory biomarkers”, “pro-inflammatory biomarkers”, “inflammatory factors”, “inflammatory cytokines”, “C Reactive Protein”, “tumor necrosis factor-alpha”, “interleukin 6”, “interleukin 1 beta”, “oxytocin”, “Brain-derived neurotrophic factor”, “psychosocial factors”, “Visual Analog Scale”, “pressure pain threshold”, “McGill pain questionnaire”, “Short-Form 36”, “Chronic Pain Grade scale”, “Pittsburgh Sleep Quality Index”, “sleep quality”, “HADS-A”, “HADS-D”, “CERQ”, “FABQ”, “catastrophism”, and the following MESH descriptors: “low back pain”, “nonspecific low back pain”, “physical activities”, “body mass index”, “inflammatory biomarkers”, “immune system”, and “psychosocial factors”. These terms were combined using the Boolean operators “AND/OR” to refine the search.

### 2.2. Eligibility Criteria

The studies included in this review had to meet the following criteria: (a) randomized clinical trials from 2019 to the present; (b) freely available or pay-to-access; (c) male or female participants up to 65 years of age; (d) subjects with nonspecific low back pain; (e) exercise, regardless of modality, as one of the main treatments for managing low back pain; (f) RCTs published in English or Spanish; (g) studies accessible in full text; and (h) those with a score equal to or greater than 7 on the PEDro scale.

Studies aimed at preventing low back pain were excluded, as well as studies on the following: (a) pregnant women, (b) individuals with conditions (e.g., infections, tumors, or rheumatoid arthritis) causing low back pain; (c) those with liver, heart, lung, or renal insufficiency, or severe tumors; (d) individuals with a history of mental illness or cerebrovascular disease; (e) individuals with a neurological disease or degenerative musculoskeletal disease; and (f) minors.

### 2.3. Data Extraction

Initially, the reviewers J.G.N. and V.B. were responsible for selecting the articles independently. They performed a screening based on the title and abstract of each study to select those relevant to the proposed research question and which aligned with the established criteria and descriptors. Duplicate studies were excluded, as well as those that, after reading the abstract or full text, were not related to the research question. In case of discrepancies, consensus was reached through discussion. In the next phase, the selected studies were analyzed in full text by the reviewers to decide which would be finally included in the review. A detailed examination was conducted to identify the studies that met the selection criteria outlined in the previous section. The entire process is documented in the flowchart according to PRISMA guidelines for the reporting of systematic reviews. For data extraction, group members worked independently and then compiled the selected articles to create a table containing the necessary data (authors, year of publication, title, objectives, study design, participants, outcome variables, intervention, comparison, results, and conclusions) for understanding and interpretation. Finally, if necessary, in the event that there was disagreement in the inclusion or exclusion of articles, a protocol was designed so that another two researchers (G.R. and J.H.V.) [20] would act as decision makers.

### 2.4. Quality Assessment

The methodological quality assessment was conducted using the PEDro Scale [21]. This analytical tool has proven to be a valid and reliable instrument for measuring the methodological quality of intervention clinical trials. It consists of 11 items, each scored with one point, allowing the evaluation of whether randomized clinical trials have sufficient internal validity (criteria 2–9) and enough statistical information to make their results interpretable (criteria 10–11). These parameters were assessed by the researchers, and all disagreements were resolved until consensus was reached.

### 2.5. Risk-of-Bias Assessment

The risk-of-bias analysis of the randomized clinical trials was conducted independently by investigators J.G.N and V.B. using the Cochrane Risk-of-Bias tool for randomized trials (RoB 2.0) [22]. The results were subsequently discussed and consolidated. This tool assesses the methodological quality of a clinical trial, scoring the presence of specific biases as yes (=1), no (=0), or unclear (=0) for each criterion. Based on the final scores, a low risk of bias indicates minimal likelihood of significant distortion of results, an unclear risk reflects some doubts about reliability, and a high risk denotes reduced confidence in the findings.

## 3. Results

### 3.1. Study Selection

**Figure 1:**
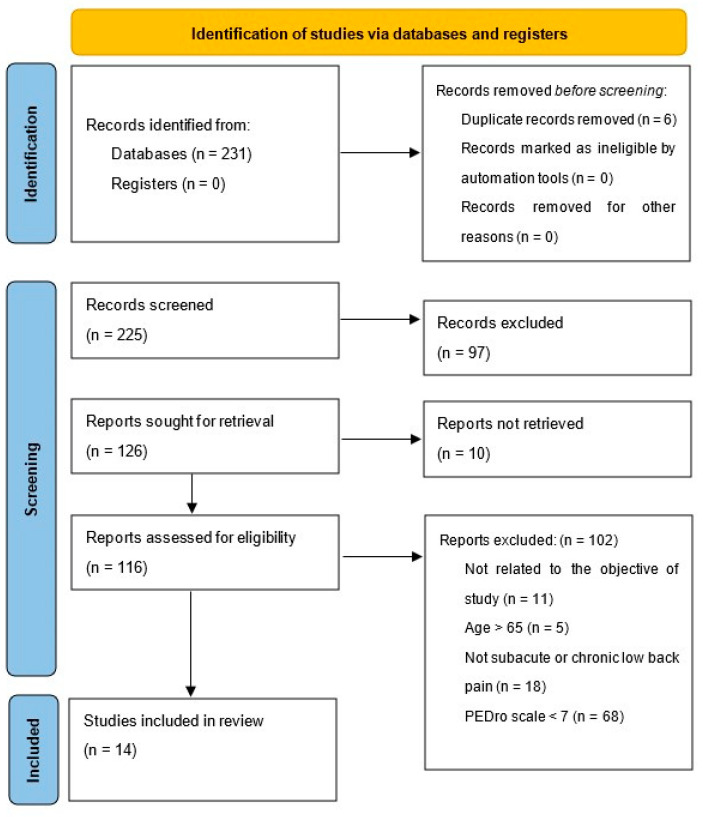
Study selection process.

From an initial corpus of 231 studies identified via searches in the PubMed, PEDro, CINAHL, Cochrane, and SCOPUS databases, six duplicates were removed. Title and abstract screening subsequently excluded 98 studies for irrelevance. A detailed full-text evaluation led to the further exclusion of 127 studies due to reasons including irrelevance to the study topic, exceeding the age limit, unavailability of the full text, nonspecificity regarding low back pain types, and subthreshold PEDro scores. Ultimately, 15 studies met the inclusion criteria for the review [23,24,25,26,27,28,29,30,31,32,33,34,35,36,37]. The flow diagram in Figure 1 delineates the selection process.

### 3.2. Characteristics of the Selected Studies

All the studies were conducted on populations with NSLBP; specifically, of the 15 studies, all were conducted on subjects with chronic pain except for one in which participants presented with subacute pain [36]. Additionally, none of the studies confirmed that the diagnosis was made following an MRI. Regarding whether patients were on pharmacological treatment, this was not specified in any of the included studies. A total of 1338 patients, aged between 18 and 65 years, were evaluated. Each of the 15 included articles assessed pain intensity as an outcome variable, although only two studies used it as the sole outcome measure [23,24]. In addition to pain, 11 articles primarily focused on the biopsychosocial factors related to NSLBP, addressing variables such as pressure pain, disability, functionality, quality of life, medication use, depression, kinesiophobia, and fear avoidance [25,26,27,28,29,30,31,32,33,34]. Four studies also included physical factors, such as hip flexibility, balance, overall flexibility, proprioception, muscle thickness, abdominal muscle strengthening, exercise capacity, core muscle activation time, and physical performance [33,34,36,37]. Only one study reported results on inflammatory biomarkers [31].

All the studies implemented some form of exercise as an intervention. Two studies focused on stretching exercises [25,27], four used strength training [28,29,32], one employed walking [26], one used sensorimotor training [23], two incorporated motor control exercises [27,32], three utilized high-intensity aerobic exercise [30,33,34], one implemented postural reeducation [23], two applied Pilates or core stabilization techniques [24,36], and one used vibration platforms [37]. All studies included in this review were conducted in a center, with the exception of Turci et al. (2023) [27], which involved self-administered exercises performed at home. However, this study did not specify the monitoring method used to assess participants’ adherence to the established protocol. Detailed characteristics of the studies are presented in Table 1.

#### 3.2.1. Effect of Exercise on Pain Intensity

There is high-quality methodological evidence and an uncertain risk of bias suggesting that graded sensorimotor retraining reduces pain intensity in patients with NSLBP, although further research is needed to evaluate its generalization [34]. On the other hand, Silva et al. (2020) [24], in a randomized clinical trial (RCT) with high methodological quality, where pain was measured daily before and after each session using the Numerical Pain Rating Scale, noted that performing Pilates more frequently during the week did not result in greater improvement for NSLBP. However, the results showed that 94.6% and 93.2% of patients in Pilates group 2 improved their pain by 30% and 50%, respectively, as did 91.9% of patients in Pilates group 1 and Pilates group 3. Moreover, 71.6%, 77%, and 78.4% of patients achieved complete pain relief in Pilates group 1, Pilates group 2, and Pilates group 3, respectively. Patients in group 1 started with a pain intensity of 3.9 and ended treatment with 1.2 (at 6 weeks), group 2 reduced from 4.5 to 0.6 (12 weeks), and group 3 from 4.8 to 0.5 (18 weeks). The following sections evaluate the reduction in pain intensity alongside the other factors studied in the review.

#### 3.2.2. Effect of Exercise on Psychosocial Factors

High-quality methodological evidence, with uncertain risk of bias, indicates that core stability and strengthening exercises, as well as stretching, improve pain intensity, disability, balance, and quality of life [25]. Suh et al. (2019) [24], in a high-quality RCT with uncertain risk of bias, found that stabilization exercises and walking reduced pain intensity and disability in patients with NSLBP, both in daily life and physical activities. Turci et al. (2023) [27], in a high-quality RCT with low risk of bias, concluded that both self-stretching exercises and motor control exercises have very similar effects on pain intensity, disability, fear avoidance, perceived effect, and flexibility, making both exercise methods useful for improving these variables. Hrkac et al. (2022) [28] observed, in a high-quality RCT, that supervised group exercise therapy combined with education (SET) was more effective compared to conventional therapy (pharmacological therapy and advice to stay active) in reducing disability, increasing quality of life, and reducing fear of performing activities after the intervention and 3 months later. Combined group exercise therapy and education (GA) had a greater effect at the 6-month follow-up.

Hernández-Lucas et al. (2023) [29], in a high-quality RCT with low risk of bias, demonstrated that strengthening and flexibility exercises, along with anatomical education and healthy lifestyle advice, performed over 14 sessions in 8 weeks, improved the Visual Analog Scale (VAS), the Roland Morris Disability Questionnaire, and the Tampa Kinesiophobia Scale compared to the control group that did not engage in any activity. Thanks to the RCT by Pinho et al. (2023) [30], with high methodological quality and low risk of bias, we know that aerobic interval training does not increase pain intensity or sensitivity.

There is high-quality methodological evidence and low risk of bias that global postural reeducation (GPR) provides greater pain relief compared to stretching [31]. Teychenne et al. (2019) [32], in a high-quality RCT, aimed to assess the influence of exercise on depression in patients with NSLBP, finding that participants who performed low-dose motor control exercises and manual therapy, or moderate-dose general strength and conditioning exercises, showed small reductions in depressive symptoms during the 6-month study period. However, these changes did not differ between the intervention groups.

Verbrugghe et al. (2019) [33] and Verbrugghe et al. (2020) [34] conducted two high-quality RCTs with low risk of bias investigating the influence of high-intensity training. In both trials, it was concluded that high-intensity exercises can be considered for the treatment of people with NSLBP, as they reduce both pain and disability and improve functionality.

#### 3.2.3. Effect of Exercise on Physical Factors

Hlaing et al. (2021) [36], in a high-quality RCT with low risk of bias, concluded that core stabilization exercises are superior to strengthening exercises for improving proprioception, balance, and muscle thickness, also producing greater improvement in NSLBP among participants. Verbrugghe et al. (2019) [33] and Verbrugghe et al. (2020) [34], in two high-quality RCTs with low risk of bias, evaluated the influence of high-intensity training, comparing different training modalities. These methods included high-intensity cardiorespiratory interval training combined with general high-intensity resistance training (HITSTRE), high-intensity core strength training (HITSTAB), a combination of high-intensity general resistance and core strength training (HITCOM), and mobility exercises (HITMOB). In both trials, it was concluded that high-intensity exercises, in any modality, lead to improvements in physical performance and strength in patients with NSLBP. Zheng et al. (2021) [37], in a high-quality RCT with low risk of bias, concluded that exercise on a whole-body vibration (WBV) platform improves activation of the multifidus, transverse abdominis, and internal oblique muscles compared to a control group without vibration, effectively reducing anticipatory delays in core muscle activation in patients with NSLBP.

#### 3.2.4. Effect of Exercise on Inflammatory Biomarkers

The only study that met the inclusion criteria and mentioned inflammatory biomarkers was by Matos et al. (2020) [31]. In this high-quality RCT with low risk of bias, urinary levels of hydroxyproline (HP), a non-essential amino acid involved in the repair and regeneration of connective tissue (31), were evaluated, showing that treatment with global postural reeducation (GPR) decreases HP levels [40]. However, no RCTs meeting the inclusion criteria were found that investigated the effects of exercise on the target biomarkers of this review, such as IL-6, TNF-α, IFN-γ, IL-1β (inflammatory cytokines), and C-reactive protein.

### 3.3. Methodological Quality Assessment

The methodological quality of the studies included in this review was generally good, with an average total score of 7.8. The studies conducted by the authors [25,26,34] were those with the lowest methodological quality, scoring 7 points on the scale.

In relation to the individual items on this scale, it is important to note that the greatest errors occurred in the blinding of subjects and therapists in these fifteen articles. Additionally, in the studies by Kim and Yim (2020) and Suh et al. (2019) [25,26], errors were made by not reporting the results for all subjects who received treatment or were assigned to the control group. Moreover, blinding of the subjects and therapists was not used in any of the studies in this review.

Finally, in the study by Verbrugghe et al. (2020) [34], it was found that not all evaluators who measured at least one key outcome were blinded. However, the later items on the scale, such as overall outcomes or measurements of subjects, were present in all articles. The methodological quality assessment is represented in the corresponding table in Table 2.

### 3.4. Risk-of-Bias Assessment

The risk-of-bias assessment conducted with the RoB 2.0 tool revealed that nine randomized clinical trials were categorized as having a “low risk” of bias, indicating robust experimental controls and reliable findings. Six studies were identified as having “some concerns” due to issues such as inadequate blinding and incomplete reporting. Importantly, no studies were classified as having a “high risk” of bias, highlighting an overall reliable evidence base.

However, specific studies, including Silva et al. (2020) [24], Hrkac et al. (2022) [28], and Teychenne et al. (2019) [32], demonstrated the highest levels of bias, mainly related to deviations from intended interventions, accounting for a 26.65% error rate.

No significant bias was identified in relation to selection, missing data, or outcome measurement. However, reporting bias was flagged as unclear in studies by Bagg et al. (2022) [23], Kim and Yim (2020) [25], and Suh et al. (2019) [26]. A detailed summary of the risk of bias assessment is provided in Table 3, offering an overview of the distribution of bias across the studies. The risk of bias assessment is shown in the corresponding table in Table 3.

## 4. Discussion

The primary objective of this systematic review was to assess the effectiveness of exercise as a treatment for NSLBP, with a particular focus on its impact on biopsychosocial and physical variables, as well as inflammatory biomarkers. The available evidence supports exercise-based interventions as beneficial, particularly in reducing pain intensity and pressure pain thresholds, as well as improving various biopsychosocial outcomes, such as depression, disability, functionality, quality of life, medication use, kinesiophobia, and fear avoidance [25,26,27,28,29,30,31,32,34]. However, no single type of exercise has been identified as the most effective for all aspects of nonspecific low back pain (NSLBP), largely due to the heterogeneity across studies. Certain approaches, nonetheless, demonstrate specific advantages in targeted areas: global postural reeducation (GPR), stabilization exercises, and high-intensity training appear particularly effective for reducing pain and enhancing quality of life, while central stabilization exercises and whole-body vibration platforms show efficacy in improving physical factors like proprioception and balance. These findings underscore the importance of individualizing exercise selection for NSLBP according to therapeutic goals and the patient’s condition.

Moreover, the review demonstrates that exercise positively affects physical parameters including proprioception, muscle thickness, abdominal strength, exercise capacity, core muscle activation, and overall physical performance [33,34,35,36,40]. These findings are consistent with the 2015 Low Back Pain Clinical Practice Guideline, which provides strong evidence supporting the initiation of an exercise regimen. The guideline highlights the importance of progressively structured and supervised exercise routines, emphasizing that no specific modality is superior but that strength training, endurance, coordination, and low-impact aerobic exercises can all be effective based on individual patient profiles [41]. There is growing evidence to suggest a link between gut microbiota and musculoskeletal health, where physical activity plays a significant modulatory role. Studies indicate that exercise can enhance gut microbiota biodiversity and influence its composition, promoting beneficial taxa and metabolic functions. However, intense physical exertion, especially in competitive athletes, may lead to dysbiosis, impacting inflammatory responses and overall health. These findings suggest that the gut–muscle axis, mediated by the microbiota, may play a role in musculoskeletal inflammation, an area deserving further exploration in non-competitive exercisers as well [42].

Additionally, recent research in the literature, including reviews by Grooten et al. (2022) [43] and Fernández-Rodríguez et al. (2022) [44], supports the use of various exercise modalities, such as Pilates, yoga, resistance training, and walking, in reducing pain and disability in patients with NSLBP. This aligns with findings from Clael et al. (2021) [45], which further indicate that exercise enhances muscle strength, endurance, and neuromuscular activity.

While there is substantial evidence supporting the efficacy of exercise in improving pain and biopsychosocial outcomes, the role of exercise in modulating inflammatory biomarkers in NSLBP remains underexplored. Barros Dos Santos et al. (2021) [46] hypothesized that exercise might affect cortisol levels, suggesting a complex relationship between physical activity and inflammation. However, the limited number of studies addressing inflammatory biomarkers limits the ability to draw definitive conclusions on this topic.

Overall, the results of this systematic review suggest that physical exercise should be considered a cornerstone of NSLBP management. Exercise programs tailored to individual preferences may enhance patient adherence and treatment outcomes, including reductions in pain, disability, and psychological factors such as depression and anxiety. However, the lack of conclusive evidence on the relationship between exercise and inflammatory biomarkers highlights a significant gap in the literature. For instance, Nambi et al. (2022) [47] observed improvements in inflammatory markers such as C-reactive protein and interleukin-6 following isokinetic exercise, although this study did not meet the methodological quality criteria for inclusion in this review. Similarly, Bruehl et al. (2020) [48] found that aerobic exercise activated endogenous opioid mechanisms, which contributed to pain relief, but their study focused on a population outside of NSLBP.

### 4.1. Future Research Directions

Future studies should aim to conduct RCTs with higher methodological rigor, particularly regarding blinding of evaluators and the inclusion of diverse populations across variables such as age, sex, and comorbidities. Moreover, research should focus on understanding the effects of exercise on inflammatory biomarkers in NSLBP using well-designed studies. Investigating long-term outcomes and their impact on quality of life and psychological well-being in NSLBP patients should also be a priority. Additionally, the studies included in this review exhibit a range of mean ages, predominantly comprising participants aged between 35 and 55 years. Notably, there exists a significant underrepresentation of younger adults, specifically those under 34 years of age. While middle-aged and older adults are sufficiently represented, the paucity of research focusing on younger populations and older seniors underscores a critical gap in the literature. Addressing these gaps in future investigations could yield valuable insights into the effects of interventions across various age groups, thereby enhancing the relevance and applicability of findings to a more diverse demographic.

### 4.2. Practical Implications

The findings from this review have direct implications for clinical practice. Exercise-based interventions should be incorporated into the treatment protocols for NSLBP, as they not only reduce pain but also improve biopsychosocial and physical parameters. Personalizing exercise regimens based on patient preferences and capabilities can optimize adherence and improve overall therapeutic outcomes. Healthcare professionals should therefore consider prescribing a range of exercise modalities tailored to each patient’s needs.

### 4.3. Study Limitations

This review presents several limitations. One of the primary challenges is the heterogeneity in both the interventions and the populations studied, which complicates the generalization of the findings. Although all patients were diagnosed with NSLBP, the variation in comorbidities and demographic characteristics across studies adds complexity to the interpretation of the results. Moreover, differences in the design and implementation of the interventions, such as variations in exercise protocols and assessment methods, further contribute to the heterogeneity. This variability, combined with methodological differences in the included studies, limited the possibility of conducting a meta-analysis. Additionally, while stringent inclusion criteria were applied, inconsistencies in methodological quality across the studies also introduce limitations in drawing firm conclusions. For example, the absence of blinding in some trials may have introduced bias in the evaluation of outcomes. Such methodological differences, particularly in outcome measures and reporting standards, reduce the robustness of the review’s findings. Consequently, the variability across studies precludes the ability to uniformly assess the impact of exercise interventions on inflammatory biomarkers and biopsychosocial outcomes, highlighting the need for more standardized and rigorously designed future research.

## 5. Conclusions

This systematic review provides robust evidence supporting the use of exercise in any modality as an effective treatment for nonspecific low back pain. Exercise not only reduces pain but also improves biopsychosocial and physical outcomes in affected individuals. While global postural reeducation (GPR), stabilization, and high-intensity exercises are particularly effective for pain and quality of life, core stabilization and vibration exercises show benefits in proprioception and balance. However, the lack of studies examining the effects of exercise on inflammatory biomarkers indicates a critical area for future research. Future studies with stronger methodological frameworks are needed to fully understand the role of exercise in modulating inflammation and its long-term impact on NSLBP.

## Figures and Tables

**Table 1:** Characteristics of selected studies.

Authors/Year of Publication	Objective	Study Design	Participants	Outcome Variables	Intervention	Comparison	Results	Conclusions
Kim and Yim, 2020 [38]	Evaluate the impact of core stability and stretching exercises in patients with NSLBP	RCT	66 patients with chronic NSLBP, 30–65 years	Pain intensity, low back instability, hip flexibility, disability, balance, quality of life	Hip muscle stretching vs. hip muscle strengthening	Stretching group vs. strengthening group	Improvements in pain, disability, balance, quality of life	Stretching and strengthening exercises significantly improve physical function and quality of life at NSLBP
Suh, J.H. et al., 2019 [26]	Compare individualized lumbar stabilization exercises with walking exercises	RCT	48 patients with chronic low back pain	VAS for low back pain, medication use, Oswestry disability index, depression	Lumbar stabilization and walking exercises	Flexibility Exercise, WE, SE, SWE	Pain reduction in all physical activities	SE and WE are recommended for their benefits in relieving pain and improving muscular endurance
Bagg et al., 2022 [23]	Estimate the effect of graded sensorimotor re-education on pain intensity	RCT	276 adults with nonspecific chronic low back pain	Pain intensity on a scale of 0 to 10	12 graduated sensorimotor retraining sessions	Care control group with simulated procedures	Significant improvement in pain intensity	The intervention improved pain intensity, with more research needed to assess generalizability
Turci et al., 2023 [27]	Compare self-administered stretching exercises with motor control exercises	RCT	100 people with chronic NSLBP	Intensity of pain, disability, fear avoidance, global perceived effect, flexibility	Stretching vs. motor control	Stretch effectiveness versus motor control	No significant differences in pain and disability	Self-administered stretching exercises as effective as motor control
Hrkac et al., 2022 [28]	Evaluate the effects of a supervised exercise program on pain and functionality in NSLBP	RCT	180 adults with chronic NSLBP	Levels of pain, functionality, quality of life, depression, and anxiety	Strengthening and flexibility exercises	Control group receiving treatment as usual	Significant improvements in pain, function, and quality of life	Supervised specific exercise program effective for improving pain and function in patients with NSLBP
Hernández-Lucas et al., 2023 [29]	Evaluate the effects of a Back School-based program on nonspecific low back pain in adults	RCT	40 patients with nonspecific chronic low back pain	Pain, disability, quality of life and kinesiophobia	8-week program including 14 practical exercise sessions and 2 theory sessions on anatomy and healthy lifestyles	Control group who maintained their usual lifestyle	Significant improvements in pain, disability, physical components of quality of life, and kinesiophobia in the experimental group	The Back School-based program is effective in improving low back pain, disability, and physical quality of life, and reducing kinesiophobia
Pinho et al., 2023 [30]	Compare interval and continuous aerobic exercise with no exercise at all	RCT	69 patients with nonspecific chronic low back pain	Intensity and sensitivity of pain to pressure (PPT)	1 set of interval aerobic exercise, 1 group continuous aerobic	Control group with no intervention	No increase in pain intensity and tenderness with 15 min of aerobic interval training	Interval aerobic exercise does not increase pain intensity or sensitivity
Matos et al., 2020 [31]	Assess pain, flexibility, and hydroxyproline levels in patients with NSLBP undergoing GPR	RCT	39 patients with nonspecific chronic low back pain	Pain, flexibility, and hydroxyproline	GPR treatment	Stretching treatment	GPR group reduced HP levels and improved flexibility more than SG	Both interventions were effective in the treatment of low back pain. However, the GPR method showed better responses than stretching
Teychenne et al., 2019 [32]	Examine the feasibility of common intervention protocols on depressive symptoms in chronic LBP	RCT	40 patients with nonspecific chronic low back pain	Depressive symptoms as measured by the CES-D scale 10	General strength and motor control exercises with manual therapy	Changes in depressive symptoms between the groups	Small reductions in depressive symptoms were observed in both groups over 6 months, with no significant differences between interventions	Both forms of exercise are useful in reducing depressive symptoms in this population
Hlaing et al., 2021 [36]	Compare the effects of core stabilization and strengthening exercises in subacute NSLBP	RCT	36 patients with nonspecific subacute low back pain	Proprioception, balance, muscle thickness, pain-related outcomes	Core stabilization exercises	Strengthening exercises	Improvements in proprioception, balance, and muscle thickness; pain relief in core stabilization group	Stabilization of the upper core to strengthen in improving proprioception, balance, and pain reduction
Verbrugghe et al., 2019 [33]	Compare a high-intensity training program with a moderate-intensity program on disability, pain, and functionality in people with NSLBP	RCT	38 patients with chronic NSLBP	Disability, pain, functionality, exercise capacity, and strength	12 weeks, 24 sessions, 1.5 h per session in high-intensity training	Moderate intensity group	Differences in favor of HIT were found for MODI, VO2max, and cycling time Improvements were found in both groups in numerical pain rating scale, VO2max performance scale, cycling time, and muscle strength	High-intensity training is a feasible, well-tolerated, and effective treatment for NSCLCBP, showing greater improvements in disability and exercise capacity compared to moderate-intensity training
Zheng et al., 2021 [39]	Determine the effect of whole-body vibration (WBV) exercises on core muscle activation in NSLBP	RCT	40 patients with nonspecific chronic low back pain	Core muscle activation time measured by sEMG	Exercises on an oscillating platform for WBV groups	Control group without vibration	Improvements in MF and TrA/IO muscle activation time in the WBV group	WBV effectively reduces core muscle anticipatory delay in patients with NSLBP
Silva et al., 2020 [24]	Analyze whether different weekly frequencies of Pilates can accelerate pain reduction in patients with NSLBP	RCT	222 patients with nonspecific chronic low back pain	Intensity of pain	3 groups: Pilates once a week; twice a week; and 3 times a week	Among the different groups	There were no differences between the different weekly Pilates frequencies	Different weekly frequencies of Pilates did not accelerate pain improvement in patients with non-specific chronic low back pain
Verbrugghe et al., 2020 [34]	Determine the efficacy of different high-intensity training modalities in NSLBP	RCT	80 patients with nonspecific chronic low back pain	Pain level, functional disability, and physical performance	4 intervention groups: - high-intensity general resistance training - high-intensity core strength training - a combined high-intensity general resistance and core strength program - mobility exercises	Comparison between different types of training	HIT improves CNSLBP rehabilitation	No differences between groups were found. High-intensity cardiorespiratory interval training improves CNSLBP rehabilitation outcomes when performed with other HIT exercise modes or mobility exercises

**Table 2:** Assessment of methodological quality.

Author and Year/Criteria PEDro	1	2	3	4	5	6	7	8	9	10	11	Total
Kim and Yim, 2020 [25]	+	+	+	+	−	−	+	+	−	+	+	7/10
Suh, J.H. et al., 2019 [26]	+	+	+	+	−	−	+	+	−	+	+	7/10
Bagg et al., 2022 [23]	+	+	+	+	−	−	+	+	+	+	+	8/10
Turci et al., 2023 [27]	+	+	+	+	−	−	+	+	+	+	+	8/10
Hrkac et al., 2022 [28]	+	+	+	+	−	−	+	+	+	+	+	8/10
Hernández−Lucas et al., 2023 [29]	+	+	+	+	−	−	+	+	+	+	+	8/10
Pinho et al., 2023 [30]	+	+	+	+	−	−	+	+	+	+	+	8/10
Matos et al., 2020 [31]	+	+	+	+	−	−	+	+	+	+	+	8/10
Teychenne et al., 2019 [32]	+	+	+	+	−	−	+	+	+	+	+	8/10
Hlaing et al., 2021 [36]	+	+	+	+	−	−	+	+	+	+	+	8/10
Verbrugghe et al., 2019 [33]	+	+	+	+	−	−	+	+	+	+	+	8/10
Zheng et al., 2021 [37]	+	+	+	+	−	−	+	+	+	+	+	8/10
Silva et al., 2020 [24]	+	+	+	+	−	−	+	+	+	+	+	8/10
Verbrugghe et al., 2020 [34]	+	+	+	+	−	−	−	+	+	+	+	7/10

**Table 3:** Risk-of-bias assessment (RoB 2.0).

Articles/Domains	D1	D2	D3	D4	D5	Overall
Kim and Yim, 2020 [25]	+	+	+	+	−	−
Suh, J.H. et al., 2019 [26]	+	−	+	+	−	−
Bagg et al., 2022 [23]	+	+	+	+	−	−
Turci et al., 2023 [27]	+	+	+	+	+	+
Hrkac et al., 2022 [28]	+	−	+	+	+	−
Hernández-Lucas et al., 2023 [29]	+	+	+	+	+	+
Pinho et al., 2023 [30]	+	+	+	+	+	+
Matos et al., 2020 [31]	+	+	+	+	+	+
Teychenne et al., 2019 [32]	+	−	+	+	+	−
Hlaing et al., 2021 [36]	+	+	+	+	+	+
Verbrugghe et al., 2019 [33]	+	+	+	+	+	+
Zheng et al., 2021 [37]	+	+	+	+	+	+
Silva et al., 2020 [24]	+	−	+	+	+	−
Verbrugghe et al., 2020 [34]	+	+	+	+	+	+

D1: Random selection bias; D2: Bias due to reduction in planned interventions; D3: Bias due to missing data; D4: Outcome measurement bias; D5: Bias in the selection of the reported outcome.

## Data Availability

Data are contained within the article.
